# Increased susceptibility to new-onset atrial fibrillation in diabetic women with poor sleep behaviour traits: findings from the prospective cohort study in the UK Biobank

**DOI:** 10.1186/s13098-024-01292-1

**Published:** 2024-02-27

**Authors:** Siwei Chen, Zhou Liu, Shaohua Yan, Zhongyan Du, Wenke Cheng

**Affiliations:** 1 Department of Cardiovascular Medicine, Nanchang People’s Hospital (The Third Hospital of Nanchang), Jiangxi, China; 2 Department of Geriatric Medicine, The Fifth People’s Hospital of Huai’an, The Affiliated Huai’an Hospital of Yangzhou University, Huai’an, China; 3 Department of Cardiology, The Fifth People’s Hospital of Huai’an, The Affiliated Huai’an Hospital of Yangzhou University, Huai’an, China; 4 Department of Cardiology, Nanfang Hospital, Southern Medical University, Guangzhou, China; 5 School of Basic Medical Sciences, Zhejiang Chinese Medical University, Hangzhou, China; 6 Key Laboratory of Blood-stasis-toxin Syndrome of Zhejiang Province, Zhejiang Engineering Research Center for “Preventive Treatment” Smart Health of Traditional Chinese Medicine, Hangzhou, 310053 China; 7 Medical Faculty, University of Leipzig, Liebigstr 27, 04103 Leipzig, Germany

**Keywords:** Behaviour sleep traits, Atrial fibrillation, Diabetes, Sleep duration, UK biobank

## Abstract

**Background:**

Diabetic individuals often encounter various sleep-related challenges. Although the association between sleep duration and atrial fibrillation (AF) have been explored, the association of other sleep traits with the incidence of AF remains unclear. A comprehensive understanding of these traits is essential for a more accurate assessment of sleep conditions in patients with diabetes and the development of novel AF prevention strategies.

**Methods:**

This study involved 23,785 patients with diabetes without any pre-existing cardiovascular disease, drawn from the UK Biobank. Sleep behaviour traits examined encompassed sleep duration, chronotype, insomnia, snoring and daytime sleepiness. Sleep duration was categorised into three groups: low (≤ 5 h), proper (6–8 h) and long (≥ 9 h). We assessed associations using multivariate Cox proportional risk regression models. Furthermore, four poor sleep behaviours were constructed to evaluate their impact on the risk of new-onset AF.

**Results:**

Over a mean follow-up period of 166 months, 2221 (9.3%) new cases of AF were identified. Short (hazard ratio (HR), 1.28; 95% confidence interval (CI) 1.10–1.50) and long sleep durations (HR 1.16; 95% CI 1.03–1.32) consistently exhibited an elevated risk of AF compared to optimal sleep duration. Early chronotype, infrequent insomnia and daytime sleepiness were associated with 11% (HR 0.89; 95% CI 0.82–0.97), 15% (HR 0.85; 95% CI 0.77–0.95) and 12% (HR 0.88; 95% CI 0.81–0.96) reduced risk of new-onset AF, respectively. However, no significant association was found between snoring and the incidence of AF (HR 0.99; 95% CI 0.91–1.07).

**Conclusions:**

In diabetic populations, sleep duration, chronotype, insomnia and daytime sleepiness are strongly associated with AF incidence. An optimal sleep duration of 6–8 h presents the lowest AF risk compared to short or long sleep duration. Additionally, poor sleep patterns present a greater risk of new-onset AF in women than in men.

**Supplementary Information:**

The online version contains supplementary material available at 10.1186/s13098-024-01292-1.

## Introduction

Sleep is a cornerstone of health and quality of life, playing a significant role in both metabolic and cardiovascular health [1]. It serves as a natural recovery phase for the body and mind, during which conscious activities are diminished, enabling various physiological recovery processes to occur [2]. Contemporary shifts in lifestyle and work habits have significantly altered sleep patterns [3]. While the importance of sleep is widely acknowledged, its significance is often underestimated, with some regarding reduced sleep as a symbol of dedication and productivity [4]. Nowadays, the health implications of sleep have risen to the level of a public health concern. Studies report that disrupted or insufficient sleep can lead to metabolic problems, trigger mild inflammatory reactions and increase sympathetic activity, all of which would increase the susceptibility to atrial fibrillation (AF) [5–7]. AF, a common cardiac disorder, affects nearly 60 million individuals worldwide and contributes to over 8 million disability-adjusted life years [8]. Its prevalence continues to increase in developed countries, propelled by risk factors such as population ageing, obesity and diabetes. Projections suggest that by 2050, the US will have between 6 and 12 million patients with AF, and by 2060, Europe's patient count will escalate to approximately 17.9 million, exerting additional strain on healthcare systems [9].

Diabetes, a widespread chronic condition, affects approximately 537 million adults globally [10]. Individuals with diabetes often face various sleep challenges compared to their non-diabetic counterparts [11]. Up to a third of those with diabetes grapple with sleep disorders, often stemming from issues like nocturia, peripheral neuropathy and restless leg symptoms [12]. These challenges can result in fragmented and shallow sleep, adversely affecting their overall quality of life. While a substantial body of research has investigated the relationship between sleep duration and cardiovascular disease, our understanding of other sleep behaviour traits, such as insomnia, snoring and daytime sleepiness, remains limited [13–15]. An understanding of these sleep behaviour traits could provide a more comprehensive assessment of sleep conditions in individuals with diabetes, offering clearer directions for future research and treatments. Furthermore, the relationship between sleep behaviour traits and the risk of AF in this population remains unclear. Therefore, the present study aims to investigate the relationship between sleep behaviour traits and the risk of new-onset AF in individuals with diabetes, offering new insights into AF prevention.

## Methods

### Study design and population

The UK Biobank, established between 2006 and 2010, is a comprehensive health database encompassing data from over 500,000 individuals aged between 40 and 69 years. These participants underwent touch-screen questionnaires, engaged in verbal interviews, underwent physical measurements and contributed biological samples at one of the 22 assessment centres located in England, Scotland and Wales. A detailed description of the study’s design and methods can be found in a previous publication [16].

In this study, a total of 34,542 individuals with diabetes completed questionnaires on sleep behaviour. After excluding participants with pre-existing AF, other arrhythmias and cardiovascular disease, 26,864 remained. Further exclusions were applied to pregnant participants (n = 4), individuals with cancer (n = 2838), and those lost to follow-up (n = 60). To minimise causality bias, patients diagnosed with AF within 2 years of follow-up (n = 177) were also excluded. Ultimately, the analysis included 23,785 participants.

### Assessment of sleep behaviour traits

The UK Biobank collected the baseline sleep data from each participant through a questionnaire. These sleep-related questions were carefully curated by international sleep experts and drew from questionnaires used in previous observational studies, clinical trials and population surveys [17, 18]. The questionnaire design aimed to strike a balance between exploring sleep patterns in-depth and minimising participant burden. Sleep behaviour traits assessed included sleep duration, chronotype, insomnia, snoring and daytime sleepiness.

1. Sleep Duration: Participants reported their average 24-h sleep duration, including naps. Their sleep duration was then categorised as short (≤ 5 h), proper (6–8 h) or long (≥ 9 h).
2. Chronotype: This metric distinguishes between participants with a propensity for morning or evening activities, classified as either a ‘definite morning type’ or a ‘definite evening type’. Morning types typically commence their day early and exhibit peak alertness in the morning, while evening types tend to stay up late, with peak alertness manifesting in the evening. Furthermore, participants were subsequently classified as either an early chronotype (definitely a 'morning' person or more a 'morning' than an 'evening' person) or a non-early chronotype.
3. Insomnia: Participants were evaluated for insomnia symptoms based on the frequency of sleep onset difficulties or nocturnal awakenings, categorised as ‘never/rarely’ or ‘often’ insomnia.
4. Snoring: Assessment of snoring relied on participants’ self-reported sleep habits and feedback from their spouses or close relatives regarding their snoring habits, grouped as ‘never/rarely snore’ or ‘frequent snorers’.
5. Daytime sleepiness: Participants were asked about the frequency of unintentional dozing during daily activities, classified as ‘never/rarely’ or ‘often’ daytime sleepiness. The detailed survey questions are presented in Additional file 1: Table S1.

### Assessment of AF

The UK Biobank integrated multiple data sources, including death registries, primary care records, hospitalisation records and self-reports, to develop diagnostic algorithms for specific health conditions. Diagnosis of AF was based on code I48 in the International Classification of Diseases, Tenth Revision (ICD-10). In this study, the date of the first record labelled as code I48 constituted the initial diagnosis date of AF for each participant. Data were continuously updated for all participants, with 1 April 2023 marking the end of the study’s follow-up period.

### Definition of diabetes

Patients with diabetes were identified using two strategies. Those with diagnosed diabetes were identified based on their self-diagnosis reports, medication use and hospital records. For those with undiagnosed diabetes, diagnostic criteria established by the American Diabetes Association were applied: a fasting blood glucose concentration of 126 mg/dL (7.0 mmol/L) or higher or a baseline HbA1c value of 6.5% (48 mmol/mol) or above [19].

### Assessment of other variables

Demographic and medical information—including age, gender, ethnicity, smoking and drinking habits, history of chronic diseases and medication use—were gathered using a touch-screen questionnaire. The Townsend Deprivation Index, an aggregate measure of poverty, was included, with higher scores indicating lower socioeconomic status. This index is derived from factors such as unemployment rates, car and homeownership rates and the extent of household crowding [20]. Lipid and glucose levels were evaluated using enzymatic assays on the Beckman Coulter AU5800 instrument (Beckman Coulter (UK), Ltd). HbA1c levels were measured using high-performance liquid chromatography on a Bio-Rad Variant II Turbo analyser (Bio-Rad Laboratories, Inc.). Body mass index was calculated from height and weight measurements taken during the participants’ initial assessment centre visit. Blood pressure readings were obtained using an automated sphygmomanometer or a manual sphygmomanometer when necessary. Hypertension was identified based on participants’ self-reports, primary care records, hospital admissions and antihypertensive drug use.

### Statistical analysis

Descriptive statistics were employed, with categorical variables presented as counts and percentages, and continuous variables as means with standard deviations. Differences among multiple groups for categorical and continuous variables were analysed using the chi-square test and one-way ANOVA, respectively. The Kaplan–Meier method and log-rank test assessed the cumulative hazard of AF with different sleep behaviour traits during follow-up. Cox proportional hazard models were used to assess the association between different sleep behaviour traits and AF risk, with results expressed as hazard ratios (HR) and 95% confidence intervals (CI). In multivariate Cox regression, potential predictors included baseline characteristics differing among multiple groups. Considering the possibility of overfitting, a variance inflation factor (VIF) was employed to quantify the degree of multicollinearity between variables, and variables with a VIF ≥ 10 were excluded [21]. Additionally, the incidence rate of AF (per 1000 person-years) across different sleep behaviour traits was also assessed.

Subgroup assessments explored the relationship between sleep behaviour traits and AF risk across various demographic characteristics and health factors, and intergroup interactions were also calculated. Restricted cubic spline curves (RCS) were plotted to visualise the relationship trend between sleep duration and risk of new-onset AF. For missing categorical variables, they were coded as missing values, whereas missing continuous variables were estimated using mean values. Detailed information on missing variables is presented in Additional file 1: Table S2. Multiple imputations were applied for missing variables, generating five data sets through the predictive mean matching algorithm and Markov chain Monte Carlo methods for sensitivity analysis [22]. Additionally, death before AF onset was considered a competing event, and a competing risk model was constructed as a sensitivity analysis [23].

All statistical analyses were conducted using R (version 4.2.0), with a significance threshold set at a two-sided p value of less than 0.05.

## Results

Among the 23,785 individuals with diabetes, the mean age was 58.5 ± 7.5 years, with 13,561 (57%) being men. Based on the distribution of sleep duration across the cohort, participants were categorised into short, proper, and long sleep duration groups. Table 1 details the baseline characteristics for each group and the differences among them. There were no significant differences between the three groups in terms of blood glucose, diastolic blood pressure and lipid-lowering medication use. Other characteristics differed significantly between the three groups, and these variables were considered potential confounders to be adjusted for in the regression models. During variable selection, total cholesterol showed strong collinearity with sleep behaviour traits (VIF ≥ 10). Therefore, cholesterol was excluded from subsequent regression models.

**Table 1:** Participants' baseline information categorized by sleep duration

Sleep duration	Total	≤ 5 h	6–8 h	≥ 9 h	*P*-value
Number	23,785	1717	19,554	2514	
Age	58.5 (7.4)	57.3 (7.5)	58.4 (7.4)	60.1 (7.3)	< 0.001
Men (%)	13,561 (57.0%)	942 (54.9%)	11,230 (57.4%)	1389 (55.3%)	0.020
White (%)	20,617 (86.7%)	1390 (81.0%)	17,014 (87.0%)	2213 (88.0%)	< 0.001
Townsend Deprivation Index	− 0.6 (3.4)	0.7 (3.7)	− 0.8 (3.3)	− 0.5 (3.4)	< 0.001
Blood glucose	7.7 (3.0)	7.8 (3.2)	7.7 (3.0)	7.8 (3.2)	0.481
HbA1_C_	51.2 (14.4)	52.2 (14.6)	51.0 (14.3)	52.2 (14.7)	< 0.001
HDL	1.3 (0.3)	1.2 (0.3)	1.3 (0.3)	1.2 (0.3)	0.005
LDL	3.0 (0.9)	3.0 (0.9)	3.0 (0.9)	2.9 (0.9)	< 0.001
Triglyceride	2.1 (1.2)	2.2 (1.3)	2.1 (1.2)	2.2 (1.2)	< 0.001
Diastolic blood pressure	82.8 (9.8)	82.8 (9.8)	83.1 (9.8)	82.6 (9.9)	0.307
Systolic blood pressure	142.3 (17.6)	141.2 (17.2)	142.3 (17.6)	142.5 (17.6)	0.017
TC	4.9 (1.2)	4.9 (1.2)	4.9 (1.2)	4.8 (1.2)	< 0.001
Body mass index	30.8 (5.9)	32.1 (6.2)	30.6 (5.8)	31.5 (6.2)	< 0.001
Current drinker (%)	20,355 (85.7%)	1360 (79.6%)	16,918 (86.7%)	2077 (82.7%)	< 0.001
Current smoker (%)	2562 (10.8%)	272 (15.9%)	2018 (10.4%)	272 (10.9%)	< 0.001
Hypertension (%)	13,435 (56.5%)	1050 (61.2%)	10,833 (55.4%)	1552 (61.7%)	< 0.001
Antihypertensives (%)	6931 (29.1%)	502 (29.2%)	5628 (28.8%)	801 (31.9%)	0.006
Lowering lipid drugs (%)	7913 (33.3%)	536 (31.2%)	6508 (33.3%)	869 (34.6%)	0.076
*Other sleep behaviour traits*					
Early chronotype (%)	13,007 (54.7%)	944 (55.0%)	10,838 (55.4%)	1225 (48.7%)	< 0.001
Never/rarely insomnia (%)	5456 (22.9%)	161 (9.4%)	4628 (23.7%)	667 (26.5%)	< 0.001
No self-reported Snoring (%)	11,704 (49.2%)	885 (51.5%)	9637 (49.3%)	1182 (47.0%)	0.013
No self-reported daytime sleepiness (%)	15,771 (66.3%)	1014 (59.1%)	13,275 (67.9%)	1482 (58.9%)	< 0.001

*HDL-C*, High-density lipoprotein cholesterol; *LDL-C*, low-density lipoprotein cholesterol; *TC*, total cholesterol; *TG*, triglyceride; *HbA1c*, glycosylated hemoglobin

### Sleep behaviour traits and risk of new-onset AF

**Fig. 1:**
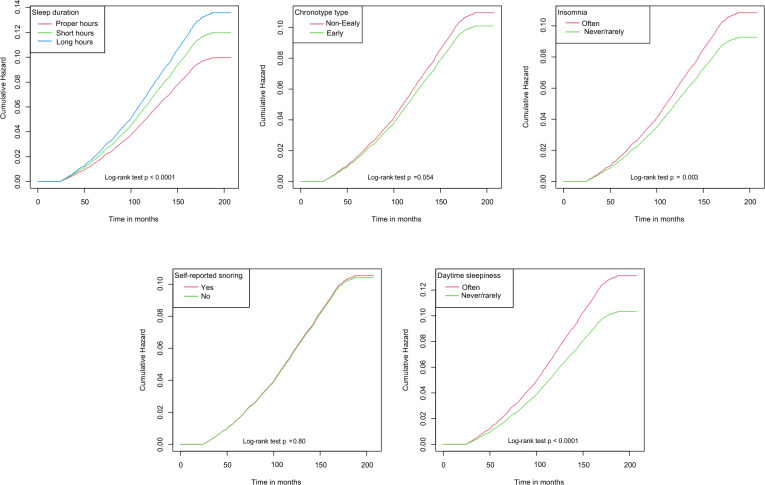
Kaplan–Meier survival curves for the cumulative hazard of new-onset AF for five sleep behaviour traits. Patients who developed AF within two years of follow-up were excluded from analysis

During a mean follow-up of 166 months (follow-up interval: 24.4–207.4), 2221 (9.3%) new cases of AF occurred. Among the five sleep behaviour traits, proper sleep duration (*p* < 0.001), early chronotype (*p* = 0.054), never/rarely insomnia (*p* = 0.003) and daytime sleepiness (*p* < 0.001) were associated with a lower cumulative hazard of AF, as shown by Kaplan–Meier curves (Fig. 1). However, snoring status was not significantly associated with the cumulative hazard of AF (*p* = 0.80).

In the short, proper and long sleep duration groups, the incidence rates of AF were 7.7, 6.42, and 8.72 per 1000 person-years. The unadjusted Cox regression models revealed that both short (HR 1.20; 95% CI 1.03–1.40) and long (HR 1.36; 95% CI 1.21–1.54) sleep durations were associated with an increased risk of AF compared to the proper sleep duration group (Table 2). After adjusting for age and sex (Model 1), this association remained consistent. Further adjustments for potential confounders (Model 2), both short (HR 1.28; 95% CI 1.10–1.50) and long (HR 1.16; 95% CI 1.03–1.32) sleep durations showed a consistently elevated risk trend for AF. Additionally, in Model 3, which incorporated other sleep behaviour traits, the increased risk associated with both short and long sleep durations persisted.

**Table 2:** Association between sleep behaviour traits and risk of atrial fibrillation in patients with diabetes mellitus

Sleep behaviour traits	N	Case	Person-years	Incidence rate^†^	Crude Model 1		Multivariate Model 1		Multivariate Model 2		Multivariate Model 3	
					HR (95% CI)	p value	HR (95% CI)	p value	HR (95% CI)	p value	HR (95% CI)	p value
*Sleep duration*												
Short hours	1717	182	23,651.7	7.70	1.20 (1.03–1.40)	0.018	1.33 (1.14–1.55)	< 0.001	1.28 (1.10–1.50)	0.002	1.25 (1.07–1.47)	0.004
Proper hours	19,554	1739	270,985.8	6.42	1.0		1.0		1.0		1.0	
Long hours	2514	300	34,399.9	8.72	1.36 (1.21–1.54)	< 0.001	1.21 (1.07–1.37)	0.002	1.16 (1.03–1.32)	0.017	1.15 (1.02–1.30)	0.027
Early chronotype	13,007	1171	179,930.2	6.51	0.92 (0.85–1.00)	0.054	0.88 (0.81–0.95)	0.002	0.89 (0.82–0.97)	0.006	0.89 (0.82–0.97)	0.007
Never/rarely insomnia	5456	452	75,702	5.97	0.85 (0.77–0.95)	0.003	0.82 (0.74–0.91)	< 0.001	0.85 (0.77–0.95)	0.003	0.86 (0.78–0.96)	0.027
Never/rarely Snoring	11,704	1087	161,905.3	6.71	0.99 (0.91–1.07)	0.797		–		–		–
Never/rarely daytime sleepiness	22,441	2067	310,620.8	6.65	0.79 (0.67–0.93)	0.004	0.77 (0.66–0.91)	0.002	0.88 (0.81–0.96)	0.005	0.82 (0.69–0.97)	0.021

^†^Incidence rate = Number of incident cases/person-years × 1000

Multivariate Model 1adjusted age and sex

Multivariate Model 2 additionally adjusted age, sex, race, body mass index, Townsend deprivation index, high-density lipoprotein cholesterol, low-density lipoprotein cholesterol, triglyceride, glycosylated hemoglobin (HbA1c), systolic blood pressure, hypertension, antihypertensives, current smoker and drinker

Multivariate Model 3 additionally adjusted other sleep behavior traits including chronotype, insomnia, snoring, daytime sleepiness

Among the other sleep behaviour traits assessed early chronotype, never/rarely insomnia, never/rarely snoring and never/rarely daytime sleepiness groups were associated with an incidence of 6.51, 5.97, 6.71 and 6.65 per 1000 person-years, respectively (Table 2). In both unadjusted and multivariable models, early chronotype, never/rarely insomnia and daytime sleepiness were significantly associated with a lower risk of AF (*p* < 0.05; Table 2). In Model 2, compared to the non-early chronotype group, the early chronotype group exhibited an 11% (HR 0.89; 95% CI 0.82–0.97) reduced risk of AF. Similarly, the groups with never/rarely insomnia and daytime sleepiness displayed a 15% (HR 0.85; 95% CI 0.0.77–0.95) and 12% (HR 0.88; 95% CI 0.81–0.96) reduced risk of new-onset AF, respectively. However, there was no significant association between snoring and the incidence of AF (HR 0.99; 95% CI 0.91–1.07).

**Fig. 2:**
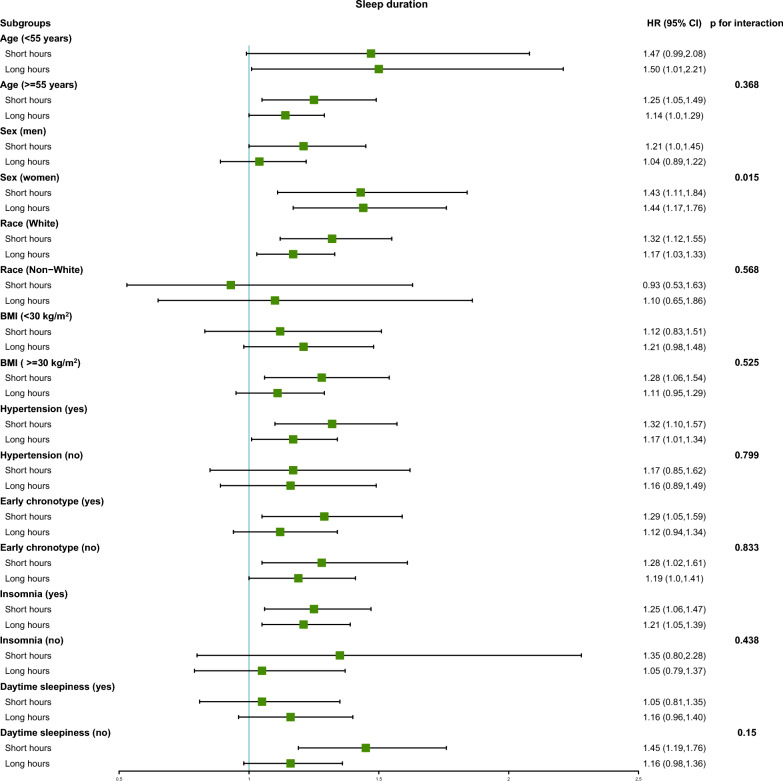
Subgroups were performed to analyze the risk of new-onset AF in each strata for short/long sleep duration. Proper sleep duration served as the reference group

In the subgroup analyses, short or long sleep durations consistently exhibited a higher risk of AF across most strata (*p* for interaction > 0.05; Fig. 2). However, a significant interaction was observed when stratified by sex (*p* for interaction = 0.015; Fig. 2). Furthermore, we evaluated the associations of early chronotype, insomnia and daytime sleepiness with AF risk in other subgroups. In most strata, these traits showed a consistent trend towards an increased risk of AF, without significant interaction effects (*p* for interaction > 0.05; Additional file 1: Figs. S1, S2, S3). Notably, interactions with sex were detected for both early chronotype and daytime sleepiness.

**Fig. 3:**
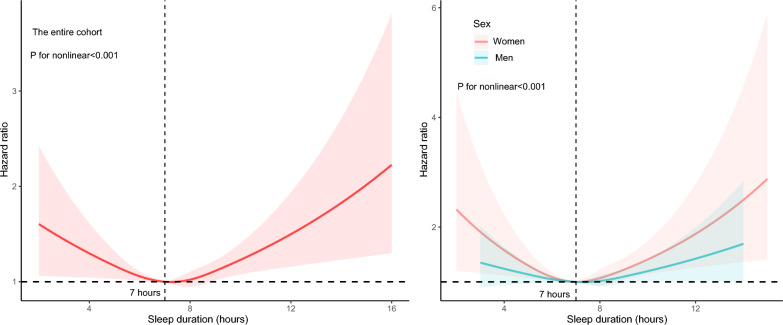
Left: Restricted cubic spline of sleep duration and risk of new-onset AF in the entire cohort. Right: Restricted cubic spline of sleep duration and risk of new-onset AF in the men and women cohorts

### Sleep duration and the risk of new-onset AF

The entire cohort exhibited a U-shaped relationship between sleep duration and the risk of new-onset AF, with 7 h of sleep associated with the lowest risk. Given the obvious differences in AF risk between the sexes, the RCS was plotted separately for men and women. As shown in Fig. 3, both men and women showed a U-shaped relationship between sleep duration and risk of AF, with the lowest risk of 7 h. Notably, the change curve for AF risk was more pronounced in women than in men.

To further refine the study, the participants were categorised into subgroups of 6, 7 and 8 h based on sleep distribution across the cohort. In a multivariate regression model, the short and long sleep groups had a 29% (HR 1.29; 95% CI 1.07–1.56) and 17% (HR 1.17; 95% CI 1.01–1.36) increased risk of AF compared to the 7-h group (Additional file 1: Table S3). However, 6- and 8-h sleep durations were not significantly associated with the risk of AF (*p* > 0.05), suggesting that 6–8 h may be the optimal range of sleep duration to reduce the risk of AF. When further adjusted for other sleep behaviour traits, this trend remained consistent.

### Different sleep behaviour patterns and risk of new-onset AF

**Fig. 4:**
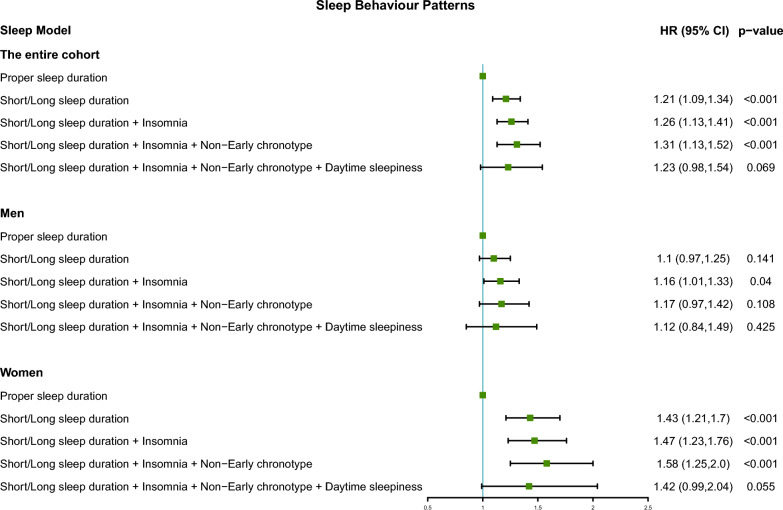
The risk of new-onset AF for four poor sleep behaviour patterns compared with proper sleep duration in the entire, men, and women cohorts

To evaluate the association of different sleep patterns with AF risk, four specific combinations of poor sleep patterns were constructed. These four patterns were: short/long sleep duration, short/long sleep duration with insomnia, short/long sleep duration with insomnia and non-early chronotype and short/long sleep duration with insomnia, early chronotype and daytime sleepiness. In the entire cohort, participants with short/long sleep durations exhibited a 21% (HR 1.21; 95% CI 1.09–1.34; Fig. 4) increased risk of new-onset AF compared to those with proper sleep durations. Upon sequentially adding insomnia and non-early chronotype to the model, the AF risk rose incrementally from 26 to 31%. However, the incorporation of daytime sleepiness did not further amplify this risk. In women, the short/long sleep duration group had a 43% (HR 1.43; 95% CI 1.21–1.7) increased risk of AF compared to those with a proper sleep duration. Similarly, as insomnia and non-early chronotype were incrementally introduced, the risk of AF increased from 47 to 58%. Nevertheless, the addition of daytime sleepiness did not further intensify this risk. Contrastingly, in the men’s cohort, AF risk increased by 16% (HR 1.16; 95% CI 1.01–1.33) in the short/long sleep duration with insomnia traits group. Moreover, for men, the risk of AF did not change significantly in other sleep patterns.

### Sensitivity analysis

A competing risks model considered death before the onset of AF as a competing event, with results consistent with the main analyses (Additional file 1: Tables S4; S5). Multiple imputations of five datasets were employed for handling missing data, with results remaining stable when these five datasets were pooled (Additional file 1: Tables S6; S7).

## Discussion

In this large prospective cohort study, we have identified significant associations between sleep behaviour traits and the risk of new-onset AF in patients with diabetes. These sleep behaviour traits include sleep duration, chronotype, insomnia and daytime sleepiness. Notably, we also observed a U-shaped relationship between sleep duration and AF risk, with the optimal range for minimising AF risk being 6–8 h of sleep per day. Importantly, our findings highlight a sex disparity in the risk of new-onset AF associated with poor sleep behaviour patterns, with a more pronounced effect in women.

To our knowledge, this is the first study to prospectively explore the relationship between sleep behaviour traits and the risk of new-onset AF in patients with diabetes. Previous observational studies and meta-analyses have indicated that both short and long sleep durations are associated with an increased risk of AF [24, 25]. Arafa et al. reported that individuals who slept ≤ 6 h or ≥ 8 h per day had an increased risk of AF compared with those who slept 7 h per day [24]. Morovatdar et al. also suggest that unhealthy sleep duration (defined as < 6 h or > 8 h) could be associated with an increased risk of AF [25]. Consistent with these studies, we similarly observed that 7 h per day was associated with the lowest AF risk in the entire cohort. However, it is essential to recognise the limitations of pinpointing sleep duration to a specific number of hours. Therefore, we further subdivided the ‘proper’ sleep duration into three groups of 6, 7 and 8 h, revealing no significant differences in AF risk among these three groups. This implies that the optimal range for reducing the risk of AF may be 6–8 h of sleep per day. Owing to the inherent limitations of observational studies, these results cannot provide evidence of causality. However, two recent Mendelian randomisation studies provide evidence for this causality, with short sleep duration being a risk factor for AF, while prolonged sleep is less likely [26, 27]. In multivariate regression models of our study, short duration sleep was more likely to develop AF, with at least a 10% increased risk compared to long-duration sleep.

Excessive sleep duration can have negative effects on both AF and cardiometabolic diseases, and may also heighten the risk of specific cardiovascular issues, such as stroke. Diabetes is a major risk factor for stroke, and AF is a common cause of embolic stroke, making the issue of sleep duration a particular concern among diabetics. Prolonged sleep duration is associated with an increased incidence of diabetes and AF, potentially due to elevated levels of inflammation, arterial stiffness, and blood pressure variability [28]. Inflammation plays a key role in atherosclerosis and cardiovascular diseases, such as stroke. The Nurses' Health Study demonstrated that prolonged sleep duration was associated with elevated levels of the inflammatory marker C-reactive protein [29]. Yoshioka et al. were the first to report an association between prolonged sleep duration and increased arterial stiffness [30]. In addition, blood pressure variability is an important indicator of arterial stiffness, and a strong predictor of stroke [31, 32]. A study by Johansson et al. involving 1,908 Finnish adults aged 41–74 years found that those sleeping more than 9 h had significantly higher blood pressure variability than those who slept for 7 h [33]. These findings highlight the importance of prolonged sleep duration as both a strong marker of stroke and a reliable risk factor, and suggest that it be emphasized in future risk classification and stroke prevention assessments [28].

In addition to sleep duration, other sleep traits such as chronotype, insomnia, snoring and excessive daytime sleepiness, have also been reported to be associated with a 10–40% increased risk of cardiovascular disease [34–37]. However, these studies have often excluded AF as an outcome. Furthermore, these studies have typically examined only a few specific sleep traits, leaving the overall relationship between sleep behaviour traits and AF risk inadequately understood. Li et al. conducted a comprehensive analysis of various sleep behaviour traits and reported that healthy sleep patterns were associated with a lower risk of AF, independent of traditional risk factors [38]. They also noted that healthy sleep patterns were particularly beneficial in populations with a lower genetic risk for AF [38]. This underscores the potential importance of healthy sleep patterns in AF prevention. Additionally, there is a growing interest in the relationship between circadian rhythms and AF. Li et al. found that early chronotype was associated with a reduced risk of AF compared to late chronotype, which is in line with our findings [38]. In subgroup analyses, we observed a significant interaction between a history of hypertension and early chronotype in the risk of AF. In hypertensive patients, an early chronotype was associated with a significantly lower risk of new-onset AF. It is well-established that persistent blood pressure fluctuations contribute to endothelial dysfunction, increased inflammatory response and structural cardiac changes, all of which are key risk factors for AF. Our observations are further corroborated by Merikanto et al., who reported that early chronotype is associated with an increased risk of hypertension [39].

Several observational studies and meta-analyses have noted that insomnia is associated with an increased risk of AF [40–42]. Wu et al. conducted a meta-analysis that revealed a 33% increased risk of AF in individuals with insomnia [42]. Moreover, Liu’s Mendelian randomisation analysis supported a causal relationship between insomnia and AF, suggesting that insomnia may increase AF risk by 13% [43]. Our study aligns with these findings, demonstrating a 15% increased risk of AF associated with insomnia. Additionally, we found that daytime sleepiness was linked to an increased risk of AF, although the literature on this topic is limited. Cang et al. reported that excessive daytime sleepiness was associated with a higher risk of AF recurrence following catheter ablation [44]. However, snoring did not appear to be associated with an increased risk of AF in our study. This discrepancy may be due to snoring not being a specific marker of sleep apnoea, as snoring is common in both normal and sleep apnoea populations. This observation is consistent with Lin et al.’s study, which also found no association between snoring and the risk of AF [45]. Obstructive sleep apnoea (OSA) is a prevalent sleep disorder that may lead to serious neurocognitive and cardiovascular sequalae in the long term [46]. Changes in cardiac structure are strongly associated with the development of AF. Currently, there is still relatively limited studies on the relationship between different OSA phenotypes and cardiovascular complications. A study by Al Oweidat K et al. found that rapid eye movement OSA was more common in patients with type 2 diabetes, and was associated with factors related to insulin resistance such as sympathetic nerve activity and increased IL-1b [47, 48]. Accurately identifying the different phenotypes of OSA is crucial for clinicians, which helps them to develop more precise and effective treatment plans for their patients, thus reducing the negative impact on cardiac structure and myocardium [48].

Notably, we observed significant sex-based differences in the association between sleep behaviour traits and the risk of new-onset AF, with poor sleep patterns having a more pronounced effect on women. Sex-related sleep differences have been previously reported in adults [49]. Women tend to report more insomnia symptoms and reduced sleep quality compared to men [50]. Studies have also noted sex differences in circadian rhythms, with women often exhibiting earlier circadian phases and shorter endogenous cycles than men [51, 52]. Women may be more susceptible to various health issues under similar sleep conditions compared to men. Cappuccio et al. reported that women had a higher risk of hypertension than men under conditions of sleep deprivation [53]. Interestingly, the risk of new-onset AF did not further increase with the addition of daytime sleepiness to poor sleep patterns. Daytime sleepiness is strongly associated with other sleep behaviour traits, often reflecting the sleep duration and insomnia of the preceding day. Moreover, we used a broad definition of daytime sleepiness, which included all self-reported instances, that may have impacted statistical efficacy. In contrast, Full et al.'s study in an older adult community-based sample did not find an increased risk of arrhythmia associated with excessive daytime sleepiness [54]. Similarly, Guo et al. did not identify a causal relationship between excessive daytime sleepiness and the onset of AF [55].

### Clinical implications

Sleep behaviour traits are interconnected, with different traits influencing and compensating for each other. A comprehensive assessment of these traits can enhance our understanding of an individual’s sleep status and associated risk factors. In this study involving patients with diabetes aged 40-6–9 years, we found that a sleep duration of 6–8 h appears to be optimal. Additionally, chronotype, insomnia and daytime sleepiness independently contribute to the risk of developing AF. Notably, the impact of unhealthy sleep patterns on AF risk is more pronounced in women compared to men. Therefore, prioritising and improving sleep quality, particularly in women, may be an effective strategy for preventing AF in the diabetic population.

### Strengths and limitations

Patients with a history of cardiovascular disease and other arrhythmias at baseline were excluded, reducing potential confounding factors. To mitigate reverse causality, participants diagnosed with AF within two years of study entry were excluded. We also addressed the sensitivity analyses, including a competing risks model and multiple imputations for missing data, which yielded results consistent with the main results, enhancing the stability of our conclusions.

Nevertheless, this study has its limitations. First, we lacked detailed information on AF subtypes, limiting our ability to conduct more specific analyses. Second, the generalizability of our findings to other populations beyond the UK Biobank cohort requires further investigation. Finally, despite our efforts to adjust for potential confounders, the impact of unaccounted residual covariates remains uncertain.

## Conclusions

In diabetic populations, sleep duration, chronotype, insomnia and daytime sleepiness are significant factors associated with the incidence of AF. Compared to short/long sleep durations, 6–8 h of sleep had the lowest risk of AF. Moreover, poor sleep patterns confer a higher risk of new-onset AF in women compared to men.

### Supplementary Information

**Additional file 1**. **Table S1. **UK Biobank touchscreen questionnaire on sleep behaviourtraits. **Table S2. **Percentage of missing values for baseline characteristics. **Table S3. **Different sleep durations and risk of atrial fibrillation. **Table S4. **Competing risk models to assess sleep behaviour traits and risk of atrial fibrillation. **Table S5. **Competing risk models for evaluating different sleep behaviourpatternsand the risk of atrial fibrillation in the entire, men and women cohorts. **Table S6. **Multivariate models to assess sleep behaviour traits and the risk of atrial fibrillation after multiple imputations. **Table S7. **Multivariate models for evaluating different sleep behaviour patterns and the risk of atrial fibrillation in the entire, men and women cohorts. **Figure S1. **Subgroup analyses were performed to examine the association between early chronotype and risk of new-onset AF (hazard ratios, 95% CIs). HR indicates the reduced risk of new-onset AF in the early chronotype group compared with the non-early-chronotype in each strata. **Figure S2. **Subgroup analyses were performed to examine the association between insomnia and risk of new-onset AF (hazard ratios, 95% CIs). HR indicates the reduced risk of new-onset AF in the non-insomnia group compared with the insomnia in each strata. **Figure S3. **Subgroup analyses were performed to examine the association between daytime sleepiness and risk of new-onset AF (hazard ratios, 95% CIs). HR indicates the reduced risk of new-onset AF in the non-daytime sleepiness group compared with the daytime sleepiness in each strata.

## Data Availability

Data can be accessed from a public and open repository. This study was conducted using the UK Biobank Resource, Application Number: 107335. Interested researchers can apply for access to the UK Biobank data at www.ukbiobank.ac.uk.
