# Supplementary material for: Increased susceptibility to new-onset atrial fibrillation in diabetic women with poor sleep behaviour traits: findings from the prospective cohort study in the UK Biobank
Source: Diabetol Metab Syndr. 2024 Feb 27;16:51. doi: 10.1186/s13098-024-01292-1 (PMC10898144; doi:10.1186/s13098-024-01292-1)

Supplement

Table S1. UK Biobank touchscreen questionnaire on sleep behaviour traits.

Table S2. Percentage of missing values for baseline characteristics.

Table S3. Different sleep durations and risk of atrial fibrillation.

Table S4. Competing risk models to assess sleep behaviour traits and risk of atrial fibrillation.

Table S5. Competing risk models for evaluating different sleep behaviour patterns and the risk of atrial fibrillation in the entire, men and women cohorts.

Table S6. Multivariate models to assess sleep behaviour traits and the risk of atrial fibrillation after multiple imputations.

Table S7. Multivariate models for evaluating different sleep behaviour patterns and the risk of atrial fibrillation in the entire, men and women cohorts.

Figure S1. Subgroup analyses were performed to examine the association between early chronotype and risk of new-onset AF (hazard ratios, 95% CIs). HR indicates the reduced risk of new-onset AF in the early chronotype group compared with the non-early-chronotype in each strata.

Figure S2. Subgroup analyses were performed to examine the association between insomnia and risk of new-onset AF (hazard ratios, 95% CIs). HR indicates the reduced risk of new-onset AF in the non-insomnia group compared with the insomnia in each strata.

Figure S3. Subgroup analyses were performed to examine the association between daytime sleepiness and risk of new-onset AF (hazard ratios, 95% CIs). HR indicates the reduced risk of new-onset AF in the non-daytime sleepiness group compared with the daytime sleepiness in each strata.

Table S1. UK Biobank touchscreen questionnaire on sleep behaviour traits.

Sleep behaviour traits	Questions	Reply
Sleep duration	About how many hours sleep do you get in every 24 hours? (Please include naps)<br>If the time you spend sleeping varies a lot, give the average time for a 24-hour day in the last 4 weeks.	Unit: hours
Chronotype	On an average day, how easy do you find getting up in the morning?	**SELECT one of 6 from**<br>1: Not at all easy; 2: Not very easy; 3: Fairly easy; 4: Very easy; -1: Do not know; -3: Prefer not to answer
	Do you consider yourself to be?	**SELECT one of 6 from**<br>1: Definitely a 'morning' person; 2: More a 'morning' than 'evening' person; 3: More an 'evening' than a 'morning' person; 4: Definitely an 'evening' person; -1: Do not know; -3: Prefer not to answer
Daytime sleepiness	How likely are you to doze off or fall asleep during the daytime when you don't mean to? (e.g., when working, reading or driving)	**SELECT one of 5 from**<br>0: Never/rarely; 1: Sometimes; 2: Often; -1: Do not know; -3: Prefer not to answer
Insomnia	Do you have trouble falling asleep at night or do you wake up in the middle of the night?<br>If this varies a lot, answer this question in relation to the last 4 weeks.	**SELECT one of 4 from**<br>1: Never/rarely; 2: Sometimes; 3: Usually; -3: Prefer not to answer
Snoring	Does your partner or a close relative or friend complain about your snoring?<br>If you are unsure, please provide an estimate or select Do not know.	**SELECT one of 4 from**<br>1: Yes; 2: No; -1: Do not know; -3: Prefer not to answer

Table S2. Percentage of missing values for baseline characteristics.

	Missing (Yes/No)	Proportion (Number / %)
Age	No	
Sex	No	
Race	No	
Townsend Deprivation Index	Yes	42 / 0.17%
Blood glucose	Yes	2867 / 12%
$HbA1_C$	Yes	1629 / 6.8%
HDL	Yes	2867 / 12%
LDL	Yes	1400 / 5.9%
Triglyceride	Yes	1368 / 5.8%
Diastolic blood pressure	Yes	60 / 0.25%
Systolic blood pressure	Yes	60 / 0.25%
TC	Yes	1327 / 5.6%
Body mass index	Yes	206 / 0.9%
Drinking status	Yes	42 / 0.2%
Smoking status	Yes	143 / 0.6%
Hypertension	No	
Antihypertensives	No	
**Sleep traits**		
Chronotype	No	
Insomnia	No	
Snoring	No	
Daytime sleepiness	No	

Table S3. Different sleep durations and risk of atrial fibrillation.

	Multivariate Model 1		Multivariate Model 2	
**Sleep duration (hours)**	HR (95% CI)	p value	HR (95% CI)	p value
≤5	1.29 (1.07-1.56)	0.007	1.27 (1.05-1.53)	0.012
6	0.96 (0.83-1.10)	0.525	0.95 (0.82-1.09)	0.442
7	reference		reference	
8	0.98 (0.87-1.10)	0.751	0.99 (0.88-1.11)	0.84
≥9	1.17 (1.01-1.36)	0.036	1.17 (1.0-1.36)	0.044

Proper sleep duration was further divided into 6, 7-, and 8-hour groups based on the results of the restricted cubic spline.

Multivariate Model 1 adjusted age, sex, race, body mass index, Townsend deprivation index, high-density lipoprotein cholesterol, low-density lipoprotein cholesterol, triglyceride, glycosylated hemoglobin (HbA1c), systolic blood pressure, hypertension, antihypertensives, current smoker and drinker.

Multivariate Model 2 additionally adjusted other sleep behaviour traits including early chronotype, never/rarely insomnia, no self-reported snoring, no frequent daytime sleepiness.

Table S4. Competing risk models to assess sleep behaviour traits and risk of atrial fibrillation. †

Sleep behaviour traits	Crude Model 1		Multivariate Model 1		Multivariate Model 2		Multivariate Model 3	
	HR (95% CI)	p value	HR (95% CI)	p value	HR (95% CI)	p value	HR (95% CI)	p value
**Sleep duration**								
Short hours	1.24 (1.06-1.44)	0.006	1.40 (1.20-1.63)	<0.001	1.34 (1.15-1.57)	<0.001	1.31 (1.12-1.53)	<0.001
Proper hours	1.0		1.0		1.0		1.0	
Long hours	1.41 (1.25-1.60)	<0.001	1.25 (1.11-1.41)	<0.001	1.19 (1.05-1.35)	0.005	1.18 (1.04-1.33)	0.009
**Early chronotype**	0.92 (0.84-1.00)	0.044	0.87 (0.80-0.95)	0.001	0.88 (0.81-0.96)	0.004	0.89 (0.81-0.96)	0.005
**Never/rarely insomnia**	0.85 (0.77-0.94)	0.002	0.81 (0.73-0.90)	<0.001	0.84 (0.76-0.93)	0.001	0.86 (0.77-0.95)	0.005
**No self-reported Snoring**	1.0 (0.92-1.08)	0.937	-		-		-	
**No frequent daytime sleepiness**	0.78 (0.66-0.91)	0.002	0.76 (0.65-0.90)	0.001	0.87 (0.80-0.95)	0.002	0.88 (0.81-0.96)	0.006

† In the competing risks model, we included death occurring earlier atrial fibrillation than as one of the outcome variables.

Multivariate Model 1adjusted age and sex.

Multivariate Model 2 additionally adjusted age, sex, race, body mass index, Townsend deprivation index, high-density lipoprotein cholesterol, low-density lipoprotein cholesterol, triglyceride, glycosylated hemoglobin (HbA1c), systolic blood pressure, hypertension, antihypertensives, current smoker and drinker.

Multivariate Model 3 additionally adjusted other sleep behaviour traits including early chronotype, never/rarely insomnia, no self-reported snoring, no frequent daytime sleepiness.

Table S5. Competing risk models for evaluating different sleep behaviour patterns and the risk of atrial fibrillation in the entire, men and women cohorts. †

**Sleep behaviour patterns**	**Entire cohort**		Men		Women	
	HR (95% CI)	p value	HR (95% CI)	p value	HR (95% CI)	p value
Proper hours	Reference		Reference		Reference	
Short/Long sleep duration	1.24 (1.12-1.38)	<0.001	1.14 (1.0-1.29)	0.051	1.48 (1.25-1.75)	<0.001
Short/Long sleep duration + Insomnia	1.31 (1.18-1.46)	<0.001	1.21 (1.05-1.39)	0.008	1.52 (1.27-1.82)	<0.001
Short/Long sleep duration + Insomnia + non-Early chronotype	1.36 (1.17-1.57)	<0.001	1.21 (1.0-1.46)	0.05	1.65 (1.30-2.08)	<0.001
Short/Long sleep duration + Insomnia + non-Early chronotype + Daytime sleepiness	1.30 (1.04-1.62)	0.02	1.19 (0.90-1.59)	0.226	1.50 (1.04-2.15)	0.028

† In the competing risks model, we included death occurring earlier atrial fibrillation than as one of the outcome variables.

Multivariate Model adjusted age, sex, race, body mass index, Townsend deprivation index, high-density lipoprotein cholesterol, low-density lipoprotein cholesterol, triglyceride, glycosylated hemoglobin (HbA1c), systolic blood pressure, hypertension, antihypertensives, current smoker and drinker.

Table S6. Multivariate models to assess sleep behaviour traits and the risk of atrial fibrillation after multiple imputations. *

Sleep behaviour traits	Multivariate Model 1		Multivariate Model 2	
	HR (95% CI)	p value	HR (95% CI)	p value
**Sleep duration**				
Short hours	1.28 (1.10-1.50)	0.002	1.25 (1.07-1.46)	0.004
Proper hours	1.0		1.0	
Long hours	1.16 (1.03-1.31)	0.018	1.15 (1.01-1.30)	0.029
**Early chronotype**	0.89 (0.81-0.96)	0.004	0.89 (0.82-0.96)	0.005
**Never/rarely insomnia**	0.85 (0.77-0.95)	0.003	0.87 (0.78-0.96)	0.009
**No self-reported Snoring**	-		-	
**No frequent daytime sleepiness**	0.85 (0.77-0.95)	0.003	0.89 (0.82-0.97)	0.011

*** Represents the pooled results of the multiple imputations (5 data sets**).

Multivariate Model 1 adjusted age, sex, race, body mass index, Townsend deprivation index, high-density lipoprotein cholesterol, low-density lipoprotein cholesterol, triglyceride, glycosylated hemoglobin (HbA1c), systolic blood pressure, hypertension, antihypertensives, current smoker and drinker.

Multivariate Model 2 additionally adjusted other sleep behaviour traits including early chronotype, never/rarely insomnia, no self-reported snoring, no frequent daytime sleepiness.

Table S7. Multivariate models for evaluating different sleep behaviour models and the risk of atrial fibrillation in the entire, men and women cohorts. *

**Sleep behaviour patterns**	**Entire cohort**		Men		Women	
	HR (95% CI)	p value	HR (95% CI)	p value	HR (95% CI)	p value
Proper hours	Reference		Reference		Reference	
Short/Long sleep duration	1.20 (1.09-1.33)	<0.001	1.10 (0.97-1.26)	0.125	1.42 (1.20-1.68)	<0.001
Short/Long sleep duration + Insomnia	1.26 (1.13-1.41)	<0.001	1.16 (1.02-1.34)	0.034	1.46 (1.22-1.74)	<0.001
Short/Long sleep duration + Insomnia + non-Early chronotype	1.31 (1.13-1.52)	<0.001	1.18 (0.97-1.43)	0.096	1.58 (1.25-2.0)	<0.001
Short/Long sleep duration + Insomnia + non-Early chronotype + Daytime sleepiness	1.23 (0.99-1.54)	0.066	1.15 (0.87-1.52)	0.340	1.40 (0.97-2.0)	0.070

*** Represents the pooled results of the multiple imputations (5 data sets**).

Multivariate Model adjusted age, sex, race, body mass index, Townsend deprivation index, high-density lipoprotein cholesterol, low-density lipoprotein cholesterol, triglyceride, glycosylated hemoglobin (HbA1c), systolic blood pressure, hypertension, antihypertensives, current smoker and drinker.

Figure S1. Subgroup analyses were performed to examine the association between early chronotype and risk of new-onset AF (hazard ratios, 95% CIs). HR indicates the reduced risk of new-onset AF in the early chronotype group compared with the non-early-chronotype in each strata.

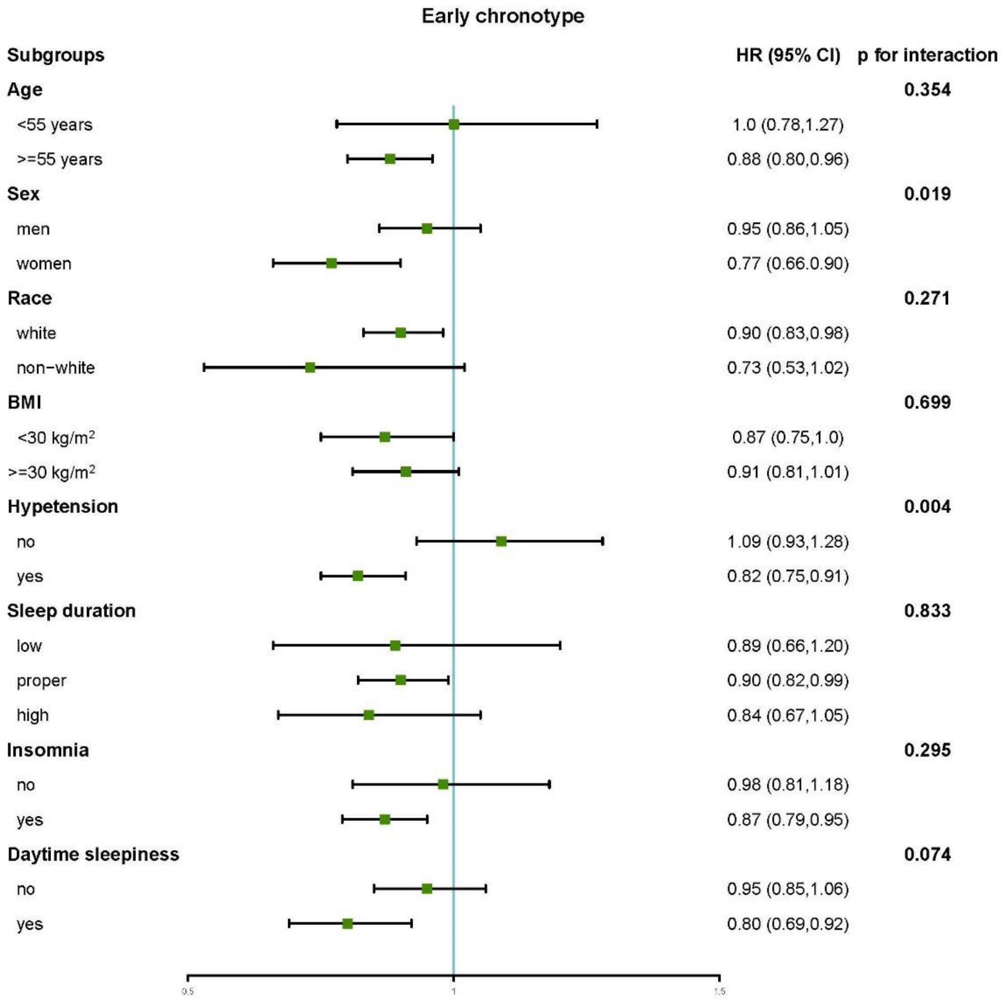

Figure S2. Subgroup analyses were performed to examine the association between insomnia and risk of new-onset AF (hazard ratios, 95% CIs). HR indicates the reduced risk of new-onset AF in the non-insomnia group compared with the insomnia in each strata.

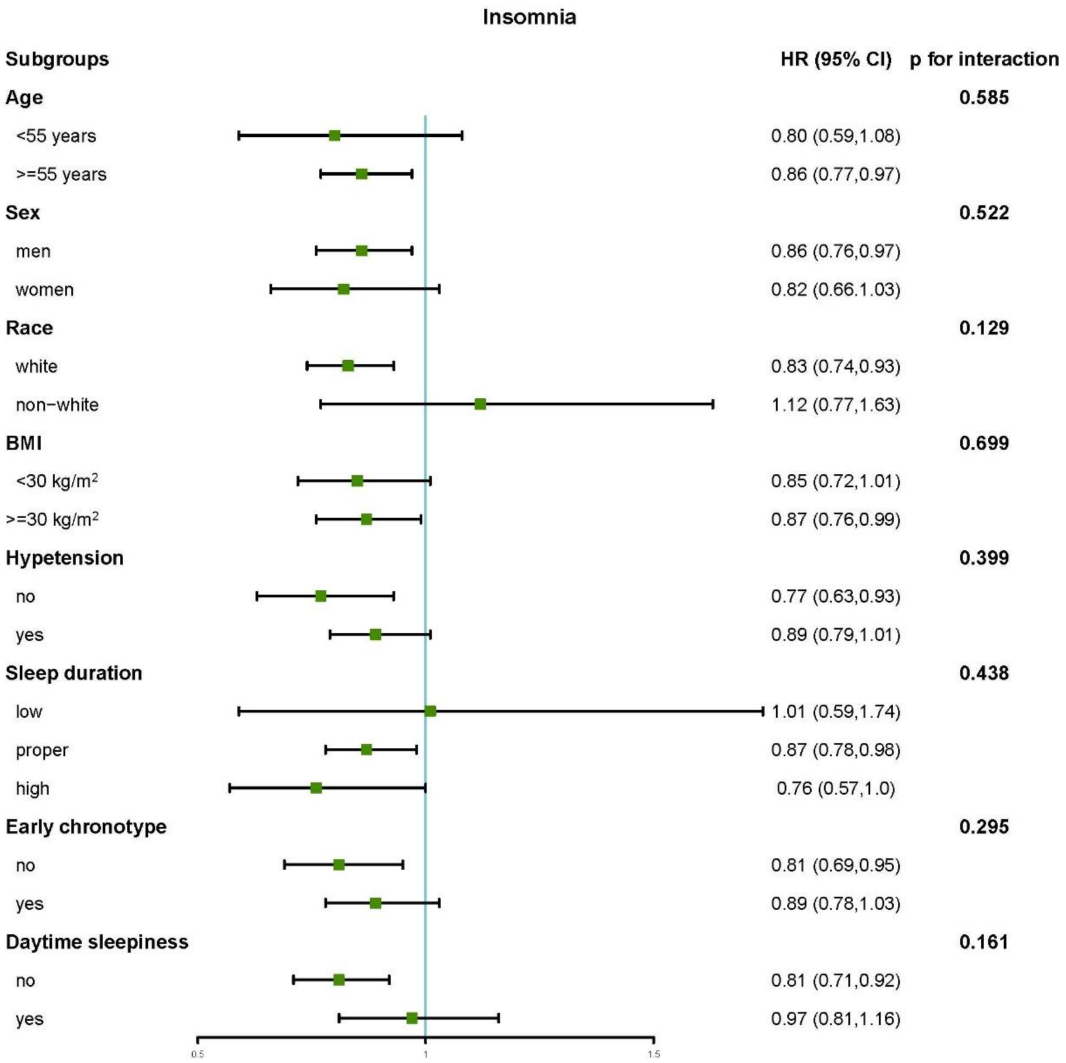

Figure S3. Subgroup analyses were performed to examine the association between daytime sleepiness and risk of new-onset AF (hazard ratios, 95% CIs). HR indicates the reduced risk of new-onset AF in the non-daytime sleepiness group compared with the daytime sleepiness in each strata**.**

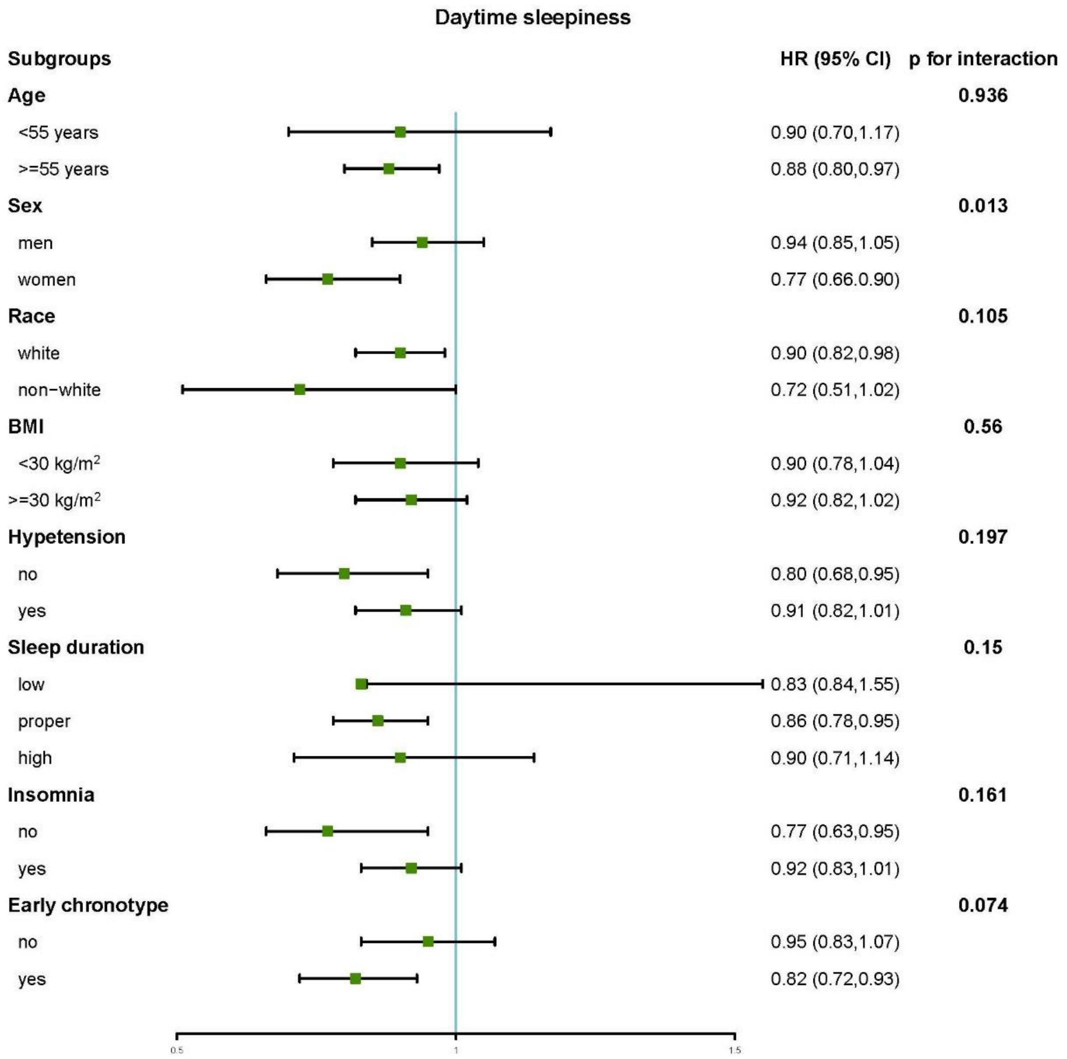

Supplement: Supplementary file 1 — Additional file 1. Table S1. UK Biobank touchscreen questionnaire on sleep behaviourtraits. Table S2. Percentage of missing values for baseline characteristics. Table S3. Different sleep durations and risk of atrial fibrillation. Table S4. Competing risk models to assess sleep behaviour traits and risk of atrial fibrillation. Table S5. Competing risk models for evaluating different sleep behaviourpatternsand the risk of atrial fibrillation in the entire, men and women cohorts. Table S6. Multivariate models to assess sleep behaviour traits and the risk of atrial fibrillation after multiple imputations. Table S7. Multivariate models for evaluating different sleep behaviour patterns and the risk of atrial fibrillation in the entire, men and women cohorts. Figure S1. Subgroup analyses were performed to examine the association between early chronotype and risk of new-onset AF (hazard ratios, 95% CIs). HR indicates the reduced risk of new-onset AF in the early chronotype group compared with the non-early-chronotype in each strata. Figure S2. Subgroup analyses were performed to examine the association between insomnia and risk of new-onset AF (hazard ratios, 95% CIs). HR indicates the reduced risk of new-onset AF in the non-insomnia group compared with the insomnia in each strata. Figure S3. Subgroup analyses were performed to examine the association between daytime sleepiness and risk of new-onset AF (hazard ratios, 95% CIs). HR indicates the reduced risk of new-onset AF in the non-daytime sleepiness group compared with the daytime sleepiness in each strata. [file 13098_2024_1292_MOESM1_ESM.pdf]
